# Cross-cultural adaptation and validation of the Norwegian pain catastrophizing scale in patients with low back pain

**DOI:** 10.1186/1471-2474-13-111

**Published:** 2012-06-22

**Authors:** Linda Fernandes, Kjersti Storheim, Ida Lochting, Margreth Grotle

**Affiliations:** 1 FORMI, Clinic for Surgery and Neurology (C1), Oslo University Hospital, Ullevaal, Oslo, Norway; 2 National Resource Center for Rehabilitation in Rheumatology, Department of Rheumatology, Diakonhjemmet Hospital, Oslo, Norway; 3 Orthopaedic Department, Clinic for Surgery and Neurology (C1), Oslo University Hospital, Ullevaal, Oslo, Norway; 4 University of Oslo, Oslo, Norway

**Keywords:** Low back pain, Pain catastrophizing, Validity, Reliability, Translation

## Abstract

**Background:**

Pain catastrophizing has been found to be an important predictor of disability and days lost from work in patients with low back pain. The most commonly used outcome measure to identify pain catastrophizing is the Pain Catastrophizing Scale (PCS). To enable the use of the PCS in clinical settings and research in Norwegian speaking patients, the PCS had to be translated. The purpose of this study was therefore to translate and cross-culturally adapt the PCS into Norwegian and to test internal consistency, construct validity and reproducibility of the PCS.

**Methods:**

The PCS was translated before it was tested for psychometric properties. Patients with subacute or chronic non-specific low back pain aged 18 years or more were recruited from primary and secondary care. Validity of the PCS was assessed by evaluating data quality (missing, floor and ceiling effects), principal components analysis, internal consistency (Cronbach’s alpha), and construct validity (Spearman’s rho). Reproducibility analyses included standard error of measurement, minimum detectable change, limits of agreement, and intraclass correlation coefficients.

**Results:**

A total of 38 men and 52 women (n = 90), with a mean (SD) age of 47.6 (11.7) years, were included for baseline testing. A subgroup of 61 patients was included for test-retest assessments. The Norwegian PCS was easy-to-comprehend. The principal components analysis supported a three-factor structure, internal consistency was satisfactory for the PCS total score (α 0.90) and the subscales rumination (α 0.83) and helplessness (α 0.86), but not for the subscale magnification (α 0.53). In total, 86% of the correlation analyses were in accordance with predefined hypothesis. The reliability analyses showed intraclass correlation coefficients of 0.74 − 0.87 for the PCS total score and subscales. The PCS total score (range 0–52 points) showed a standard error of measurement of 4.6 points and a 95% minimum detectable change estimate of 12.8 points.

**Conclusions:**

The Norwegian PCS total score showed acceptable psychometric properties in terms of comprehensibility, consistency, construct validity, and reproducibility when applied to patients with subacute or chronic LBP from different clinical settings. Our study support the use of the PCS total score for clinical or research purposes identifying or evaluating pain catastrophizing.

## Background

The Pain Catastrophizing Scale (PCS) was developed by Sullivan et al.[1], and has been widely used in the research of chronic pain and pain behavior in patients with low back pain (LBP). Pain catastrophizing, explained as an exaggerated negative orientation towards noxious stimuli [1], has been found to correlate significantly to pain severity, self-reported disability, pain behavior, fear of movement and depression in patients with low back pain (LBP) [1-5]. Furthermore, in chronic pain patients, pain catastrophizing has been identified as an important mediator to pain behavior and pain-related fear [6-8]. In the cognitive-behavioral fear-avoidance model, presented by Vlaeyen et al.[2], pain catastrophizing was found to be the strongest predictor of fear of movement. Patients who were fearful of movement performed worse in physical performance tests and were also more anxious after the test was completed [2]. Moreover, pain catastrophizing and pain behavior have been found to predict days lost from work and failure to return to work in subacute and chronic LBP patients [9-12]. It has been recommended that risk factors such as pain catastrophizing, fear of movement, and distress should be identified early and targeted in interventions specifically designed to reduce pain catastrophizing and fear [8,12,13]. A recent cohort study in patients with work-related injuries found that a reduction in catastrophic thinking and fear of movement, over a 10-week intervention period, predicted return to work [3]. Thus, pain catastrophizing can be reduced by targeted interventions and, importantly, influence return to work.

To be able to evaluate interventions targeting pain catastrophizing and investigate its significance in Norwegian LBP populations, we translated and cross-culturally adapted the English PCS [1] into Norwegian and tested the Norwegian PCS for psychometric properties in terms of validity and reproducibility in patients with LBP.

## Methods

### Study design

The study was carried out in a two-step procedure; firstly, the PCS was translated and cross-culturally adapted; and secondly, the Norwegian PCS was tested for psychometric properties in a cross-sectional design with a 1-week follow-up for test-retest.

### Translation and cross-cultural adaptation

Translation and cross-cultural adaptation of the PCS was performed according to international guidelines [14,15]. Two persons (one clinician and one philologist), whose mother tongue is Norwegian, independently translated the original PCS into Norwegian. The two Norwegian versions were synthesized into one Norwegian version before it was translated back to English. Two translators and native speaker of English, who were blinded to the original PCS, performed the back-translation. One expert committee consisting of the translators, one health professional and the researchers in our research group reviewed all translations. In a formal meeting, the committee discussed discrepancies until consensus on a prefinal version was achieved. The goal of the prefinal Norwegian PCS was that it should be as concise and easy to understand as possible. The first 4–5 patients with low back pain at each participating clinic reviewed the prefinal Norwegian PCS. None of the patients had difficulties in understanding the meaning of items or responses. Since the prefinal version was highly acceptable and easy-to-comprehend, no changes were made and the final version of the Norwegian PCS was equal to the prefinal. The English and Norwegian versions are presented in Additional file 1: Appendix.

### Participants

A total of 103 participants were recruited from different clinical settings in Oslo, Norway, between September 2008 and November 2009. Eligible participants were patients with non-specific LBP for six weeks or more prior to inclusion, aged 18 and over, and were able to speak, read and write in Norwegian. Exclusion criteria were sciatica or signs of ‘red flags’ (tumor, infection, spinal fracture, or major neurologic compromise such as cauda equina syndrome) [16]. The inclusion was performed by a clinician, mostly a physical therapists, seeing the patients at their clinic. Four patients were excluded because of signs of sciatica and nine patients did not return the baseline questionnaires without giving any reason for not participating. A total of 90 patients, 52 women and 38 men, were included; 30 patients from three physical therapy clinics (primary care), 24 patients from an outpatient rehabilitation clinic, six patients from a pain unit (university hospital), and 30 patients from an orthopaedic department (university hospital). Sixty-one patients participated in the test-retest design, of whom 60 filled in the PCS at retest. Baseline characteristics of the whole sample and the test-retest subgroup are presented in Table 1. All patients received written and oral information about the study. Signed informed consent was obtained from all patients. The study was approved by the Norwegian Regional Committee for Medical Research Ethics and the Data Inspectorate in 2008.

**Table 1:** Characteristics of the Study Sample; the Whole Sample (n = 90) and the Sub-group Participating in Test-retest (n = 60)

	***Whole Sample***	***Test-retest Sub-group***
		
Age (yrs.)	47.6 (11.7)	49.2 (11.2)
Sex (% Women)	52 (58%)	32 (52%)
Born in Norway (% yes)	80 (89%)	58 (95%)
Civil status		
Single	21 (23%)	15 (25%)
Married/cohabiting	69 (77%)	46 (75%)
Work status		
Employed	40 (44%)	24 (39%)
Sick-listed	18 (20%)	15 (25%)
Pension (disability, retired)	25 (28%)	18 (30%)
Pain localization		
Lower back only	32 (36%)	23 (38%)
Lower back and other sites	57 (63%)	38 (62%)
Pain duration		
Less than 3 months	19 (21%)	11 (18%)
3 months or more	71 (79%)	50 (82%)
Pain duration (yrs.)	9.7 (13.2)	11.0 (13.3)
Back pain Numeric Rating Scale (0–10)	4.8 (2.1)	4.9 (2.1)
Pain Catastrophizing Scale total score (0–52)	13.6 (9.2)	13.6 (8.9)
Roland Morris Disability Questionnaire (0–24)	7.6 (5.2)	7.2 (5.1)
Core Outcome Measure Index (0–10)	5.6 (1.9)	5.6 (1.8)
Fear Avoidance Beliefs Questionnaire, Physical Activity (0–24)	9.4 (5.5)	8.8 (5.3)
Fear Avoidance Beliefs Questionnaire, Work (0–42)	16.7 (11.9)	17.0 (11.2)
Hopkins Symptom Check List-25 (1–4)	1.61 (0.49)	1.64 (0.49)
EQ-5D	0.54 (0.31)	0.55 (0.29)

### Procedures and measures

The included patients filled in the PCS, sociodemographic information, and concurrent measures at the first attendance for assessment. Patients consenting to participate at the retest filled in the PCS and a global score (6-pointLikert scale) of change in LBP status between test and retest at the second attendance, preferably with a one-week interval.

#### The PCS

The PCS comprises 13 items focusing on thoughts and feelings. The original PCS was evaluated in undergraduate students and was found to be a reliable and valid measure of catastrophizing with a three factor solution; *rumination (4 items)*, ruminative thoughts, worry, and inability to inhibit pain-related thoughts; *magnification (3 items)*, magnification of the unpleasantness of pain and expectancies for negative outcomes; and, *helplessness (6 items)*, inability to deal with painful situations [1]. Patients score the 13 items on a 5-point Likert scale, ranging from 0 (*not at all*) and 4 (*all the time*), relating the items to the past painful experience. Separate sub-scores for the dimensions (range, rumination 0–16; magnification 0–12; and helplessness 0–24 points) or a total score (range, 0–52 points) can be calculated for the PCS. A higher score indicates higher pain catastrophizing. Internal missing values were replaced with mean values if the number of missing items did not exceed two items, except for analysis of data quality.

#### Concurrent measures

To assess construct validity, the PCS was compared with outcome measures widely used and evaluated, which assess physical functioning, fear avoidance, psychological well-being and distress, quality of life, and pain. The Roland Morris Disability Questionnaire (RMDQ) measures pain related physical functioning [17], Core Outcome Measures Index (COMI) is a short multi-dimensional scale measuring the five most important domains relating to low back pain [18,19], Fear Avoidance Believes Questionnaire Physical Activity subscale (FABQ-PA) and Work subscale (FABQ-W) measure fear avoidance [20], Hopkins Symptom Check List-25 (HSCL-25) measures anxiety, depression and somatization [21,22], EuroQol-5 Dimensions Index (EQ-5D) measures quality of life [23], and the Numeric Rating Scale (NRS) measures back pain [24].

### Statistical analysis

Sample size was based on the quality criteria recommended by Terwee et al.[25], who suggest a minimum of 50 patients for assessing construct validity, reproducibility, and floor or ceiling effects, and a minimum of 100 patients for assessing factor analysis and internal consistency.

Descriptive analysis included mean (SD) and number (%). Missing data and end effects were described. Floor or ceiling effects were considered to be present if more than 15% of the included sample scored the lowest or the highest score, respectively [25].

Principal components analysis [26] was used to assess the underlying structure of the PCS items. Components were extracted with an eigenvalue greater than one. Internal consistency was assessed by calculating the Cronbach’s alpha coefficient (α). A Cronbach’s alpha coefficient between 0.70 and 0.95 has been considered acceptable homogeneity [25].

The construct validation of the PCS total score was assessed by comparing the PCS for association to concurrent measures. Predefined hypotheses of association were established based on the construct of the measures. Hence, we expected measures of psychological constructs, i.e. the FABQ and the HCSL-25, to have moderate to high correlation to the PCS. Whereas measures of other constructs, i.e. the RMQD, COMI, EQ-5D, and NRS back pain, were expected to have low to moderate correlation to the PCS. All correlation coefficients, except for the EQ-5D, were expected to correlate positively to the PCS. The EQ-5D was expected to correlate negatively to the PCS, as scaling has an inverse relationship. Normality and linearity assessments were performed for the included variables. Assumption of linear relationship could not be confirmed. Hence, construct validity was assessed by the Spearman’s rank order correlation coefficients (rho). High correlations were equivalent to correlation coefficients of *rho* ≥ 0.60, moderate to *rho* <0.60 – ≥0.30, and low to *rho* < 0.30 [27]. An adequate construct validity of the PCS was achieved if 75% or more of the results were in correspondence with the predefined hypothesis [25].

Reproducibility was analyzed by reliability and agreement parameters and applied to the total score and subscales of the PCS. Main analysis was performed on the whole sample participating at test and retest. A supplementary analysis was conducted in patients reporting a stable (unchanged) status between test and retest.

Reliability was tested by applying the intraclass correlation coefficient (ICC) consistency formula [ICC_consistency_ = σ_patients_^2^/σ_patients_^2^ + σ_residual_^2^[28] and its 95% confidence interval (CI). Agreement was tested by the standard error of measurement (SEM) consistency formula [SEM_consistency_ = √ σ_residual_^2^[28]. The error variance (within people residual mean square) was extracted from an ANOVA analysis. The 95% confidence level of the minimum detectable change (MDC_95_) was calculated to evaluate the smallest within-person change in score by calculating the formula [MDC_95_ = 1.96 × √2 × SEM] [28]. The MDC_95_ has been described as a “real” change, if above measurement error and *p* < 0.05, in one individual [25]. In addition, the percentage of the MDC in relation to the maximum score was calculated. As described by Bland & Altman, scatter plots of intraindividual difference between test and retest against the grand mean [mean_test1_ + mean_test2_/2] were generated. Limits of agreement (95%) were calculated by the formula [mean difference ± 1.96 × SD_difference_[29]. The statistical package SPSS 16.0 for Windows, Release 16.0.2 (IBM® SPSS® Statistics, NY) was used for all analyses.

## Results

### Data quality

Overall, patients were able to complete the PCS without assistance. Nine patients had a total of 21 internal missing values. Missing values were spread over 12 out of 13 items. Item 1 ‘I worry all the time whether the pain will end’ had no missing value. The median PCS total score was 12 points (range, 0 to 37 points). Floor effects were seen in 12 out of 13 items, but not for PCS total score or subscales. No ceiling effects were found. Data quality is presented in Table 2.

**Table 2:** Internal missing values (n = 90 patients × number of items) and N (%) scoring the lowest or highest score on the Pain Catastrophizing Scale (n = 90). Higher score indicating higher degree of pain catastrophizing

**Pain Catastrophizing Scale**	**Range**	**Internal missing** **N (%)**	**Mean (S.D.)**	**Lowest N (%)**	**Highest N (%)**	
*Total score*	0 − 52	21 (0.02)	13.6 (9.2)	3 (3.3)	0	
*Rumination subscale*	0 − 16	7 (0.02)	5.0 (3.5)	6 (6.7)	0	
8	I anxiously want the pain to go away	0 − 4	1 (0.002)	1.9 (1.3)	16 (17.8)	9 (10.0)
9	I can’t seem to keep it out of mind	0 − 4	2 (0.005)	0.8 (0.9)	40 (44.4)	2 (2.2)
10	I keep thinking about how much it hurts	0 − 4	2 (0.005)	0.8 (0.8)	35 (38.9)	0
11	I keep thinking about how badly I want the pain to stop	0 − 4	2 (0.005)	1.6 (1.3)	20 (22.2)	9 (10.0)
*Magnification subscale*	0 − 12	4 (0.01)	2.8 (2.0)	12 (13.3)	0	
6	I become afraid that the pain may get worse	0 − 4	2 (0.007)	1.6 (1.2)	16 (17.8)	5 (5.7)
7	I think of other painful experiences	0 − 4	1 (0.004)	0.4 (0.7)	63 (70.0)	0
13	I wonder whether something serious may happen	0 − 4	1 (0.004)	0.7 (0.9)	47 (52.2)	1 (1.1)
*Helplessness subscale*	0 − 24	7 (0.02)	6.2 (4.8)	6 (6.7)	1 (1.1)	
1	I worry all the time whether the pain will end	0 − 4	0	1.5 (1.1)	15 (16.7)	4 (4.4)
2	I feel I can’t go on	0 − 4	2 (0.004)	0.9 (1.1)	43 (47.8)	1 (1.1)
3	It’s terrible and I think it’s never going to get any better	0 − 4	1 (0.002)	1.1 (1.1)	33 (36.7)	3 (3.3)
4	It’s awful and I feel that it overwhelms me	0 − 4	3 (0.006)	0.8 (1.1)	46 (51.1)	2 (2.2)
5	I feel I can’t stand it any more	0 − 4	1 (0.002)	0.8 (1.1)	52 (57.8)	3 (3.4)
12	There is nothing I can do to reduce the intensity of the pain	0 − 4	3 (0.006)	1.2 (1.0)	23 (25.6)	1 (1.1)

### Factor analysis and internal consistency

The principal components analysis revealed a three-factor structure, which accounted for 64.5% of the total variance (Table 3). The items loading on the first component (item 11, 10, 8, 9, 6) had loadings ranging from 0.57 to 0.79, the second component (item 13, 1, 7) from 0.52 to 0.89, and the third component (item 12, 4, 2, 5, 3) from 0.54 to 0.87, respectively. The first component accounted for 47.9% of the total variance, whereas component two and three accounted for 8.1% and 8.8%, respectively. The internal consistency, assessed by Cronbach’s alpha, was 0.90 for the total score and 0.86, 0.63, and 0.83 for the three subscales according to current factor structure revealed in this material. When using the same three-factor structure as in the original study, the Cronbach’s alpha for the first and third components were highest with values of 0.86 for the *helplessness* subscale and 0.83 for the *rumination* subscale. The *magnification* subscale had a Cronbach’s alpha of only 0.53.

**Table 3:** Pain Catastrophizing Scale factor structure by Principal Components Analysis with loadings (n = 90)

**Pain Catastrophizing Scale**		**Components**		
		**Rumination**	**Magnification**	**Helplessness**
11	I keep thinking about how badly I want the pain to stop	0.79		
10	I keep thinking about how much it hurts	0.78		
8	I anxiously want the pain to go away	0.72		
9	I can’t seem to keep it out of mind	0.67	0.43	
6	I become afraid that the pain may get worse	0.57	0.32	
13	I wonder whether something serious may happen		0.89	
1	I worry all the time whether the pain will end	0.49	0.61	
7	I think of other painful experiences	0.37	0.52	
12	There is nothing I can do to reduce the intensity of the pain			0.87
4	It’s awful and I feel that it overwhelms me	0.60		0.64
2	I feel I can’t go on	0.33		0.63
5	I feel I can’t stand it any more	0.57		0.61
3	It’s terrible and I think it’s never going to get any better	0.52		0.54

Extraction Method: Principal Component Analysis; varimax rotation with Kaiser normalization. Rotation converged in six iterations; values below 0.3 are suppressed. The model explained 64.7% of the total variance; component 1 explained 47.9%, component 2 = 8.1%, and component 3 = 8.8%.

### Construct validity

The PCS total score showed moderate correlation coefficients to the FABQ-PA (rho = 0.34) and HSCL-25 (rho = 0.56), as well as to the COMI (rho = 0.43), EQ-5D (rho = −0.36), and NRS back pain (rho = 0.31). Moreover, the PCS total score correlated low to the RMDQ (rho = 0.27) and FABQ-W (rho = 0.25) (Table 4). In total, 86% of the a priori hypotheses were confirmed.

**Table 4:** Predefined Hypothesis of Correlation and Spearman’s rho Coefficients for the PCS Total Score and Concurrent Measures (n = 90)

	**Correlation coefficient (rho)**	**Hypothesis confirmed yes/no**
*Moderate to high correlation*		
FABQ-PA	0.34**	Yes
FABQ-W	0.25*	No
HSCL-25	0.56**	Yes
*Low to moderate correlation*		
RMDQ	0.27*	Yes
COMI	0.43**	Yes
EQ-5D	-0.36**	Yes
NRS back pain	0.31**	Yes
*p < 0.05 (two-tailed)		
**p < 0.01 (two-tailed)		

FABQ-PA: Fear Avoidance Believes Questionnaire Physical Activity; FABQ-W: Fear Avoidance Believes Questionnaire work; HSCL-25: Hopkins Symptom Check List-25; RMDQ: Roland Morris Disability Questionnaire; COMI: Core Outcome Measures Index; EQ-5D: EuroQual-5 Dimensions Index; NRS: Numeric Rating Scale.

### Reproducibility

**Figure 1:**
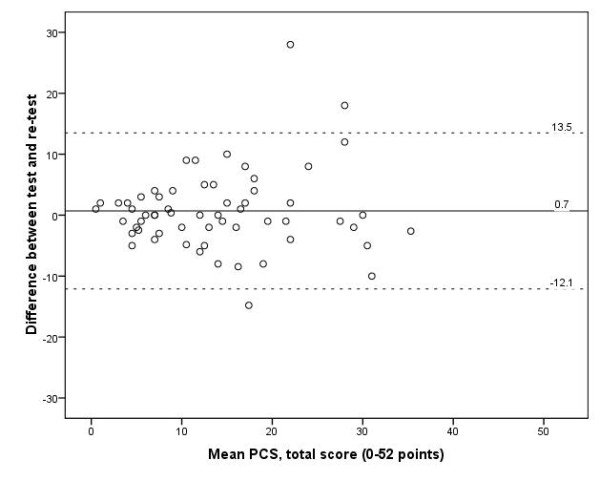
Scatter plot of intraindividual difference between test and retest against the grand mean of the total score of the Norwegian Pain Catastrophizing Scale (n = 60).

The median time between test and retest was 7 days (range, 1–30 days). The PCS total score showed a mean (SD) of 13.6 (8.9) and 14.1 (9.5) points at test and retest, respectively, and an ICC (95% CI) between tests of 0.85 (0.76, 0.91). The SEM of the PCS total score was 4.6 points and the MDC_95_ 12.8 points. Reproducibility data of the PCS total score and subscales are presented in Table 5. The mean difference between test and retest was 0.7 points, on the PCS total score, with limits of agreement of 13.5 and −12.1 points (Figure 1). Thirty-five patients scored “no change” in LBP status between test and retest, of whom 34 had filled in the PCS twice. Supplementary analyses of stable patients (n = 34) showed slightly higher ICCs and somewhat lower SEMs and MDCs (Table 6).

**Table 5:** Test-retest Statistics of the Pain Catatstrophizing Scale (n = 60)

	**Mean (S.D.)**	**Mean (S.D.)**	**ICC (95% CI)**	**SEM**	**MDC**_**95**_	**MDC%**
	**test**	**retest**				
*Total score (0–52)*	13.6 (8.9)	14.1 (9.5)	0.85 (0.76, 0.91)	4.6	12.8	24.6
*Rumination (0–16)*	5.0 (3.4)	5.2 (4.0)	0.74 (0.56, 0.84)	2.4	6.7	41.6
*Magnification (0–12)*	2.7 (2.2)	2.6 (2.3)	0.82 (0.70, 0.89)	1.2	3.5	29.0
*Helplessness (0–24)*	5.9 (4.3)	6.3 (4.1)	0.87 (0.78, 0.92)	2.0	5.6	23.3

ICC: Intraclass correlation coefficient; SEM: standard error of measurement. MDC: minimum detectable change.

SEM_consistency_ = √_Within people Residual Mean square_.

MDC_95_ = 1.96 × √2 × SEM.

MDC % = MDC as percentage of maximum sore.

**Table 6:** Test-retest Statistics of the Pain Catatstrophizing Scale in stable subjects (n = 34)

	**Mean (S.D.)**	**Mean (S.D.)**	**ICC (95% CI)**	**SEM**	**MDC**_**95**_	**MDC%**
	**test**	**retest**				
*Total score (0–52)*	14.9 (9.8)	14.2 (9.1)	0.92 (0.84, 0.96)	3.7	10.2	19.6
*Rumination (0–16)*	5.4 (3.7)	5.4 (3.9)	0.79 (0.59, 0.90)	2.2	6.2	38.8
*Magnification (0–12)*	2.9 (2.5)	2.4 (2.0)	0.89 (0.78, 0.95)	1.0	2.7	22.1
*Helplessness (0–24)*	6.5 (4.8)	6.3 (4.3)	0.91 (0.83, 0.96)	1.8	5.1	21.1

ICC: Intraclass correlation coefficient; SEM: standard error of measurement. MDC: minimum detectable change.

SEM_consistency_ = √_Within people Residual Mean square_.

MDC_95_ = 1.96 × √2 × SEM.

MDC % = MDC as percentage of maximum sore.

## Discussion

This study demonstrated that the Norwegian PCS was comprehendible, easily administered and, overall, held acceptable psychometric standards when assessed in patients with non-specific LBP recruited from different clinical settings. The PCS total score appeared to be more robust than the subscales alone. We suggest that the Norwegian PCS total score can be used in clinical settings and research in patients with non-specific LBP across different settings for purposes evaluating pain catastrophizing.

The principal components analysis supported a three-factor structure similar to the original study of Sullivan et al.[1]. Also later studies support this finding [30-32]. There were minor differences with respect to which items that loaded on the three components, however. This is also similar to what is reported in other studies that have validated the PCS [30,32]. We found a slightly better fit for factor structure with item 6 in the *rumination* subscale than in the *magnification* subscale (as in the original model) and with item 1 in the *magnification* subscale than in the *helplessness* subscale. The differences across the studies might be due to differences in patient samples. As suggested by Meyer et al.[32], the PCS scores should be reported according to the subscales and the total score as recommended by Sullivan et al. in the original study [1]. Otherwise, it will be impossible to compare the results across studies using different item solutions in the different components. The internal consistency, according to the current factor structure found in our study and the original structure, showed acceptable homogeneity (α 0.83–0.90) for the subscales *rumination* and *helplessness* and the total score. Earlier studies have shown acceptable homogeneity for the total score and all subscales in both clinical and nonclinical samples and have suggested that the PCS can be used as one score (13 items) or as three separate scores independently [1,30,31,33]. Based on the results in our study, the *magnification* subscale did not show an acceptable internal consistency (α 0.63 and 0.53) and, on the contrary to earlier studies, we cannot suggest the independent use of it. Evidence was found for construct validity, since 86% of the results in the correlation analysis were in accordance with predefined hypothesis. The hypotheses were based on existing literature of the PCS and its relation to different aspects of health, e.g. more catastrophizing thoughts has been found to be associated with more distress, disability and fear avoidance beliefs [1,5,32]. Higher associations have been found for variables measuring psychological aspects of health compared with variables measuring physical aspects of health. The PCS showed, as presumed, higher correlation coefficients to concurrent measurements of psychological constructs (e.g. distress by HSCL-25) as compared to outcomes of other constructs such as disability, pain and health related quality of life. However, the PCS correlated lower than expected to the FABQ-W and showed a lower correlation coefficient compared to one previous study (rho = 0.25 versus r = 0.61) [32]. This result might be explained by the fact that the FABQ-W has specific questions related to work and only 40 patients (44%) in our study were employed. The rate of employed in the study by Meyer et al.[32] was not described. The correlation between the PCS and pain severity was in our study found to be lower than in earlier published articles (rho 0.31 versus r = 0.41 − 0.55)[1,3-5], but in correspondence to the earlier studies the correlation coefficient was significant.

The reliability of the Norwegian PCS showed high ICCs (above 0.70) for both total score and subscales (Table 5). Hence, the Norwegian PCS has the potential to discriminate between patients with different levels of catastrophizing [28]. Though, for PCS total scores above 37 points we could not ascertain the reliability since the score ranged from 0 to 37 points in the included sample. Compared to studies including patients with LBP[31,33,34], our sample had lower mean scores (mean PCS total score 20–30 versus 14 points). One possible reason for a lower mean score in our sample may be the inclusion of patients with *subacute* LBP, as opposed to commonly seen inclusion criteria of chronic LBP only. Recalling that pain catatrophizing has been discussed as one mediator of chronicity of symptoms [6-8] and is a predictor of failure to return-to-work [9-12], pain catastrophizing thoughts has been suggested to be identified early to better target interventions [8,12]. In our sample, 21% could be defined as patients who seek health care “early” (pain duration of <3 months) and, therefore, may score lower on the PCS compared to a sample including chronic pain patients only.

Main strengths of the present study were, as far as our design allowed, the inclusion of psychometric properties for the evaluation of health status questionnaires as recommended by Terwee et al.[25]. This study is among the first to include agreement analyses for the PCS. In medical research evaluating interventions targeting pain catastrophizing thoughts, agreement properties of outcome measures estimating potential effect are of importance [28]. Our study showed that the PCS total score held superior agreement as compared to the individual subscales (Table 5 and 6). Compared to two earlier studies, the MDC_95_ in our study (12.8 points) was equal to the findings of Meyer et al. (12.8 points) [32], but higher than the findings of Georg et al. (9.1 points) [35]. The percentage of the MDC_95_ in relation to the maximum score for the PCS total score was 25%. Subsequently, an individual has to change 25% on the PCS total score between pre- and post-intervention to exceed measurement error and its 95% CI and to be classified as having a “real” change in pain catastrophizing score. To fully interpret our agreement analyses, the SEMs and MDCs need to be compared to minimum important changes, rated by patients’ opinion, which can be performed in responsiveness analyses [25]. One study of responsiveness found a high standardized response mean (SRM 1.12) for the PCS, suggesting that the PCS can capture change over time, but no analyses of minimum important changes were performed [36]. Further investigations of minimum important change of the PCS would therefore be needed. The median number of days between tests was seven days, though, the range between tests was one to 30 days. Recall bias is a potential risk for patients seeing the clinician with a short interval and change in LBP status is a risk when the time lapse between tests is long. To avoid potential recall bias, the PCS was part of a comprehensive assessment packet both at test and retest and it was filled in after attendance at both test and retest. One clear limitation in our study was that 25 of 60 patients who participated in the test-retest assessment scored “change” in LBP status between test and retest. For that reason we completed supplementary analyses of the 35 “stable” LBP patients. The reproducibility analysis of the 35 stable LBP patients showed slightly better results (compare Table 5 and 6). Another limitation was sample size. Our sample size for the principal components analysis was 90 patients, which is less than 100 or 150 participants recommended for factor analysis [25,26].

## Conclusion

The Norwegian PCS total score shows evidence for acceptable psychometric properties in terms of being fully comprehensible, internally consistent, reproducible, and comprising a valid construct when applied in patients with subacute or chronic LBP from different clinical settings, but internal consistency and agreement was questionable for the individual subscales. Our study supports the use of the PCS total score for clinical or research purposes identifying or evaluating pain catastrophizing. Further investigations of responsiveness of the PCS are suggested.

## Competing interests

The authors declare that they have no competing interests.

## Authors’ contributions

KS and MG conceptualized and designed the study. IL was responsible for the acquisition of data. LF, KS, and MG analyzed and interpreted the data. LF drafted the manuscript and KS, IL and MG have critically revised the manuscript. All authors read and approved the final manuscript.

## Pre-publication history

The pre-publication history for this paper can be accessed here:

http://www.biomedcentral.com/1471-2474/13/111/prepub

## Supplementary Material

Additional file 1: **Appendix.** Pain Catastrophizing Scale. Click here for file
