# Supplementary material for: Cross-cultural adaptation and validation of the Norwegian pain catastrophizing scale in patients with low back pain
Source: BMC Musculoskelet Disord. 2012 Jun 22;13:111. doi: 10.1186/1471-2474-13-111 (PMC3407790; doi:10.1186/1471-2474-13-111)
Supplement: Additional file 1: — Appendix. Pain Catastrophizing Scale. [file 1471-2474-13-111-S1.pdf]

APPENDIX

English PCS

Pain Catastrophizing Scale

Client No.: __________ Age: _____ Sex: M(__) F(__) Date: _______________

Everyone experiences painful situations at some point in their lives. Such experiences may include headaches, tooth pain, joint or muscle pain. People are often exposed to situations that may cause pain such as illness, injury, dental procedures or surgery.

We are interested in the types of thoughts and feelings that you have when you are in pain. Listed below are thirteen statements describing different thoughts and feelings that may be associated with pain. Using the following scale, please indicate the degree to which you have these thoughts and feelings when you are experiencing pain.

0 – not at all **1** – to a slight degree **2** – to a moderate degree **3** – to a great degree **4** –all the time

When I'm in pain …

1 I worry all the time about whether the pain will end.

2 I feel I can't go on.

3 It's terrible and I think it's never going to get any better.

4 It's awful and I feel that it overwhelms me.

5 I feel I can't stand it anymore.

6 I become afraid that the pain will get worse.

7 I keep thinking of other painful events.

8 I anxiously want the pain to go away.

9 I can't seem to keep it out of my mind.

10 I keep thinking about how much it hurts.

11 I keep thinking about how badly I want the pain to stop.

12 There's nothing I can do to reduce the intensity of the pain.

13 I wonder whether something serious may happen.

…Total

Norwegian PCS

SKALA FOR SMERTEDRAMATISERING

Alle opplever smerter på et aller annet tidspunkt i livet. Slike smerteopplevelser kan være hodepine, tannverk, ledd- og muskelsmerter. Folk er ofte utsatt for situasjoner som kan forårsake smerter, slik som sykdom, skade, tannbehandling og kirurgi.
Vi er interessert i hva slags tanker og følelser du har når du har smerter. Nedenfor står det 13 utsagn som beskriver ulike tanker og følelser som kan være forbundet med smerte. Bruk følgende skala og indiker i hvilken grad du har slike tanker og følelser når du opplever smerte.

Når jeg har smerter …		**Ikke i det hele tatt**	**Litt**	**I moderat grad**	**I stor grad**	**Hele tiden**
1	jeg er hele tiden bekymret for at smertene ikke vil gi seg	0	1	2	3	4
2	jeg føler at jeg ikke klarer å fortsette	0	1	2	3	4
3	det er forferdelig og jeg tror at det aldri vil bli bedre	0	1	2	3	4
4	det er fryktelig, og jeg føler at det overvelder meg	0	1	2	3	4
5	jeg føler at jeg ikke holder det ut lenger	0	1	2	3	4
6	jeg blir redd for at smertene skal bli verre	0	1	2	3	4
7	jeg tenker stadig på andre smertefulle opplevelser	0	1	2	3	4
8	jeg ønsker desperat at smertene skal forsvinne	0	1	2	3	4
9	det virker som jeg ikke klarer å få det ut av hodet	0	1	2	3	4
10	jeg tenker stadig på hvor vondt det er	0	1	2	3	4
11	jeg tenker stadig på hvor inderlig jeg vil at smertene skal gi seg	0	1	2	3	4
12	det er ingenting jeg kan gjøre for å redusere smertenes intensitet	0	1	2	3	4
13	jeg lurer på om noe alvorlig kan komme til å skje	0	1	2	3	4

……….Sum
